# Carryover Effects of Acute DEHP Exposure on Ovarian Function and Oocyte Developmental Competence in Lactating Cows

**DOI:** 10.1371/journal.pone.0130896

**Published:** 2015-07-08

**Authors:** Dorit Kalo, Ron Hadas, Ori Furman, Julius Ben-Ari, Yehoshua Maor, Donald G. Patterson, Cynthia Tomey, Zvi Roth

**Affiliations:** 1 Department of Animal Sciences, Robert H. Smith Faculty of Agriculture, Food and Environment, the Hebrew University, Rehovot, 76100, Israel; 2 Center of Excellence in Agriculture and Environmental Health, Robert H. Smith Faculty of Agriculture, Food and Environment, the Hebrew University, Rehovot, 76100, Israel; 3 Interdepartmental Equipment Facility, Robert H. Smith Faculty of Agriculture, Food and Environment, the Hebrew University, Rehovot, 76100, Israel; 4 AXYS Analytical Services Inc., Sidney, British Columbia, V8L 5X2, Canada; Institute of Zoology, Chinese Academy of Sciences, CHINA

## Abstract

We examined acute exposure of Holstein cows to di(2-ethylhexyl) phthalate (DEHP) and its carryover effects on ovarian function and oocyte developmental competence. Synchronized cows were tube-fed with water or 100 mg/kg DEHP per day for 3 days. Blood, urine and milk samples were collected before, during and after DEHP exposure to examine its clearance pattern. Ovarian follicular dynamics was monitored through an entire estrous cycle by ultrasonographic scanning. Follicular fluids were aspirated from the preovulatory follicles on days 0 and 29 of the experiment and analyzed for phthalate metabolites and estradiol concentration. The aspirated follicular fluid was used as maturation medium for in-vitro embryo production. Findings revealed that DEHP impairs the pattern of follicular development, with a prominent effect on dominant follicles. The diameter and growth rate of the first- and second-wave dominant follicles were lower (*P* < 0.05) in the DEHP-treated group. Estradiol concentration in the follicular fluid was lower in the DEHP-treated group than in controls, and associated with a higher number of follicular pathologies (follicle diameter >25 mm). The pattern of growth and regression of the corpus luteum differed between groups, with a lower volume in the DEHP-treated group (*P* < 0.05). The follicular fluid aspirated from the DEHP-treated group, but not the controls, contained 23 nM mono(2-ethylhexyl) phthalate. Culturing of cumulus oocyte complexes in the follicular fluid aspirated from DEHP-treated cows reduced the proportion of oocytes progressing to the MII stage, and the proportions of 2- to 4-cell-stage embryos (*P* < 0.04) and 7-day blastocysts (*P* < 0.06). The results describe the risk associated with acute exposure to DEHP and its deleterious carryover effects on ovarian function, nuclear maturation and oocyte developmental competence.

## Introduction

Environmental pollution includes a variety of widespread man-made chemicals. Some of these, e.g. phthalates, have been classified as endocrine-disruptor compounds (EDCs), as they can interfere with normal endocrine functions [1], such as in the male and female reproductive systems [2–4].

Phthalates are plasticizers used in a variety of industrial plastics applications. Given their weak binding to the plastic, phthalates readily leak into the environment and are widely distributed in the air, soil and water [5–8]; thus they can easily penetrate an organism's body. For instance, di(2-ethylhexyl) phthalate (DEHP), the most commonly used plasticizer, is rapidly hydrolyzed by lipases in the gut, liver and blood into mono(2-ethylhexyl) phthalate (MEHP), which in turn oxidized to mono(2-ethyl-5-hydroxyhexyl) phthalate (5OH-MEHP), mono(2-ethyl-5-oxohexyl) phthalate (5oxo-MEHP), mono(2-ethyl-5-carboxypentyl) phthalate (5cx-MEPP) and mono[2-(carboxymethyl)hexyl] phthalate (2cx-MMHP) [9,10].

In humans, DEHP and its metabolites have been documented in the serum, seminal plasma [11], cord blood serum [12], urine [11,13,14], and peritoneal, follicular and amniotic fluids [15–17]. To date, DEHP has been reported in bovine milk [18,19] and fat tissue [19], in ewe [20] and in swine [19,21] tissues. Being lipophilic compounds, phthalates are transferred and accumulated in the adipose tissue; they therefore have the potential to release into the circulation during periods of high fat metabolism, such as postpartum or in early lactation [22]. DEHP kinetics has been recorded for mice, rats, marmosets [23, 24], swine [21] and humans [25], with differences seen among species [21,24]. However, kinetics and precise physiological levels of DEHP and its metabolites are unknown in other domestic animals, such as lactating cows.

DEHP has been suggested as a reproductive toxicant for both humans and animals [4,26,27] and is considered the most potent toxicant among phthalates [28]. Oral administration of DEHP to female rats impairs the estrous cycle timing, inhibits ovulation in association with alteration in estradiol, progesterone, follicle-stimulating hormone (FSH) and luteinizing hormone (LH) concentrations [29,30]. Exposure of adult mice to DEHP decreases the number of primordial follicles and increases the number of primary follicles [31]. Culturing mouse antral follicles with DEHP inhibits their growth and decreases estradiol production by follicular cells [32]. In-vitro exposure of newborn mouse ovaries to DEHP reduces the number of primordial follicles and increases the number of nested germ cells [33]. In-utero exposure of female rats to high levels of DEHP during gestation increases the number of tertiary follicles undergoing atresia in the offspring [34]. Nevertheless, the effects of DEHP and its metabolites on ovarian function and oocyte developmental competence in domestic animals are less known.

In-vitro studies in bovines indicate that both DEHP and MEHP have a negative impact on oocyte maturation [35–38] and developmental competence [37]. In our previous study, we found that in-vitro exposure of bovine oocytes to 50 μM MEHP impairs nuclear and cytoplasmic maturation and further reduces embryonic development postfertilization [37,38]. Moreover, lower doses of DEHP and MEHP have been reported in the literature to alter oocyte meiotic progression [35,39,40]. Nevertheless, there is a lack of data on phthalate concentrations in lactating cows and their effects in vivo.

In the current study, we established a model to examine the dynamics of DEHP metabolite clearance in the urine, plasma and milk of lactating cows after exposure to an acute dose of DEHP (100 mg/kg per day for 3 days). In addition, we examined the carryover effects of DEHP on the growth and regression of follicles and corpus luteum by ultrasonographic monitoring through an entire estrous cycle following DEHP exposure. Follicular fluid (FF) from the preovulatory follicle was aspirated, analyzed for estradiol concentration and phthalate metabolite contents. The aspirated FF was then used as medium for in-vitro maturation of bovine oocytes. The oocytes' developmental competence, i.e., their ability to cleave into 2- and 4-cell-stage embryos and to develop to blastocysts (42 h and 7 days postfertilization, respectively) was determined. Thus our findings explore the carryover effects of DEHP on ovarian function.

## Materials and Methods

### Ethics statements

The study was conducted at the experimental dairy farm in Bet-Dagan, Israel with the permission of the Israeli Agricultural Research Organization. The study was carried out in strict accordance with the recommendations in the guide for the care and use of animals of the Israeli Institutes of health. The protocol was approved by the Institutional Animal Care and Use Committee (IACUC) of the Israeli Agricultural Research Organization (Permit Number: 296/10). Procedure for follicular aspiration, including anesthesia, was specifically approved. Cows were sedated with 2 ml xylazine hydrochloride administered (i.m) (Sedaxylan; Eurovet Animal Health BV, Bladel, Holland) and caudal epidural anesthesia was induced with 5 ml lidocaine (Esracain; Rafa Laboratories Ltd., Jerusalem, Israel). Sampling of milk, blood and urine, as well as estrus synchronization were reviewed as a part of obtaining the field permit. All efforts were made to minimize animal suffering.

### Animals

A total of nine cyclic Holstein cows were kept in an open shed with access to an adjacent yard, and ad-libitum access to a total mixed ration containing 1.37 Mcal/kg dry matter and 13.2% protein.

### Experimental design

**Fig 1:**
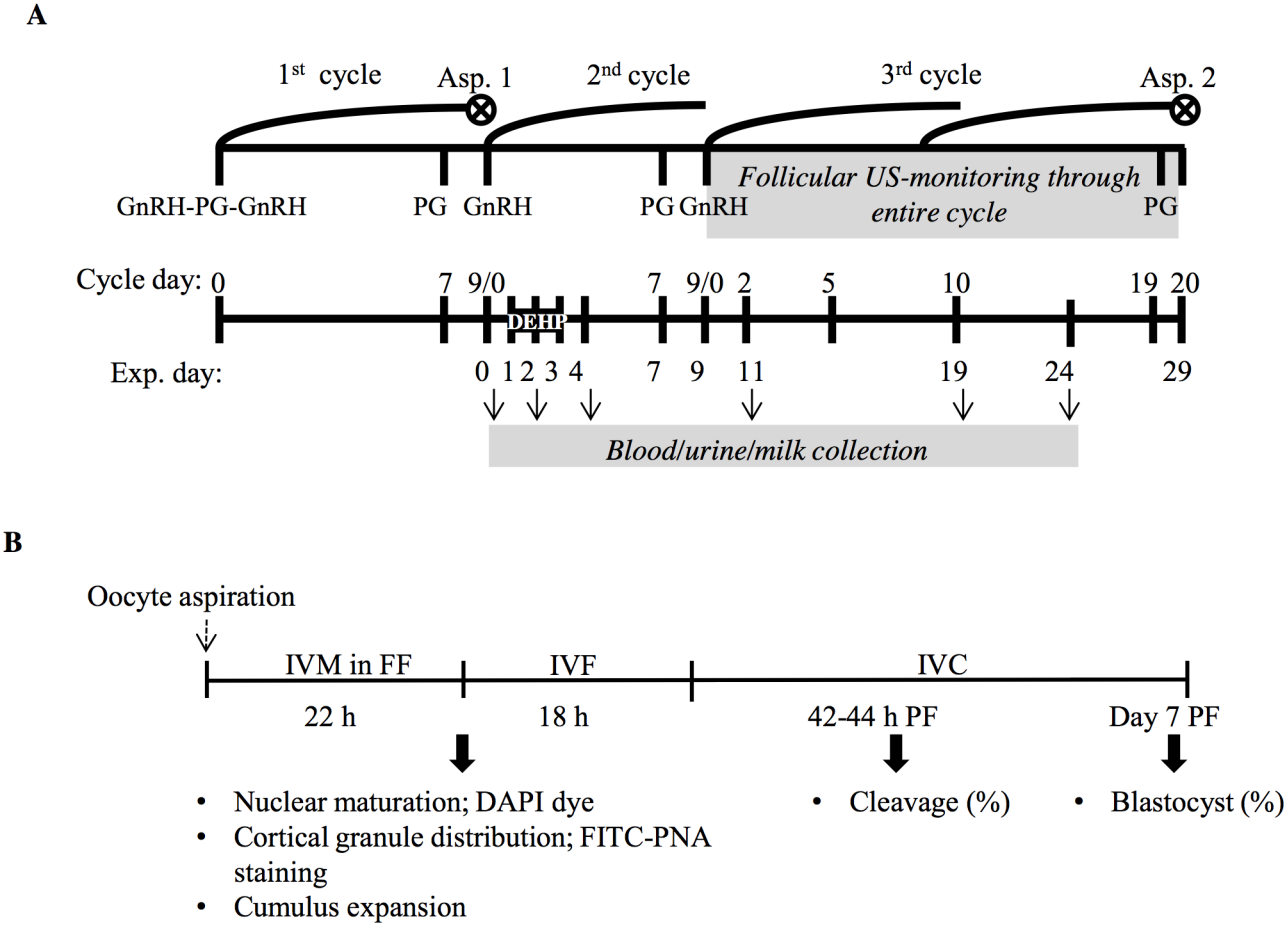
Schematic illustration of the experimental design. In the in-vivo part (A), cows were synchronized according to the ‘Ovsynch’ protocol. Cows were then divided into control (n = 5) and DEHP-treated (n = 4) groups. Samples of blood, urine and milk were collected on day 0 (before), days 2 and 4 (during) and days 11, 19 and 24 (after) DEHP administration. On day 7 of the experiment, cows were resynchronized and ovarian follicular dynamics was monitored by linear ultrasonographic scanner through the entire third synchronized cycle (days 9 to 29 of the experiment). For each experimental group, FF were pooled and analyzed for phthalate metabolite and estradiol concentrations (Asp. 1 and 2). In the second part of the study (B), FF aspirated from control and DEHP-treated cows were used as oocyte maturation medium. Cumulus oocyte complexes were aspirated and in-vitro matured in the aspirated FF (IVM in FF; 22h). Subgroups of mature oocytes were collected for examination of nuclear meiotic stages (DAPI staining), cortical granule distribution (FITC–PNA) and cumulus cell expansion. Another subgroup was in-vitro fertilized (IVF) for 18 h then in-vitro cultured (IVC) for 8 days. Oocyte developmental competence was evaluated as the proportion of oocytes that cleaved into 2- to 4-cell-stage embryos and developed to blastocysts 44 h and 7 days postfertilization, respectively.

The study consisted of two parts: in vivo and in vitro (Fig 1). In the in-vivo part (Fig 1A), cows were synchronized according to the ‘Ovsynch’ protocol: animals were injected intramuscularly (i.m.) with 2 ml gonadotropin-releasing hormone (GnRH analog; Gonabreed, Parnell Laboratories, Alexandria, NSW, Australia), followed by i.m. injection of 2.5 ml prostaglandin (PG) F_2α_ (Cloprostenol, Estroplan, Parnell Laboratories) 7 days later, and a second GnRH injection (2 ml) 48 h after PGF_2α_ administration. The day of the second GnRH injection was defined as day 0 of the first synchronized cycle. On day 7 of this cycle, cows were injected with an additional 2.5 ml PGF_2α_ and the FF of the preovulatory follicle was aspirated 30 h later (Asp. 1; Fig 1A). Cows were then injected with 2.0 ml GnRH to induce luteinization of the aspirated follicle (day 0 of the second synchronized cycle and of the experiment; Fig 1A).

Thereafter, cows were arbitrarily divided into two experimental groups and tube-fed with water (n = 5, control) or with 100 mg/kg DEHP daily for 3 days (n = 4, DEHP-treated). Samples of plasma, urine and milk from each experimental group were collected before (day 0), during (days 2 and 4) and after (days 11, 19 and 24) DEHP exposure to determine the clearance pattern of DEHP metabolites and their concentrations in each fraction (Fig 1A). For each day, group and fraction samples were pooled and stored at -20°C until analysis. Samples were then analyzed by LC–MS/MS to determine concentrations of MEHP, 5OH-MEHP, 5oxo-MEHP, 2cx-MMHP and 5cx-MEPP.

Cows were resynchronized by PGF_2α_ followed by GnRH injection on days 7 and 9 of the second cycle, respectively. Ovarian follicular dynamics was monitored by linear ultrasonography scanner through the third synchronized cycle (days 9 to 29) and blood samples for progesterone concentration analysis were collected on days 2, 5, 10 and 20 of the examined cycle. FF of the preovulatory follicles was aspirated (Asp. 2) after DEHP administration (day 29; Fig 1A). For each experimental group and for each aspiration, FFs were pooled and analyzed for phthalate metabolites and estradiol concentration.

The in-vitro part of the study (Fig 1B) was performed to examine the carryover effects of phthalates. Oocytes were collected from Holstein cow ovaries obtained from a local abattoir and matured for 22 h in the FF aspirated (Asp. 2) from control or DEHP-treated cows as previously described [41]. At the end of maturation, subgroups of oocytes from each experimental group were stained with (1) fluorescein isothiocyanate-coupled peanut agglutinin (FITC–PNA) to determine cortical granule (CG) distribution and (2) 4',6-diamidino-2-phenylindole dihydrochloride (DAPI) to determine nuclear meiotic status. Another subgroup of oocytes was in-vitro fertilized for 18 h and in-vitro cultured for 8 days. The proportion of oocytes that cleaved into 2- to 4-cell-stage embryos and developed to blastocysts was recorded 44 h and 7 days postfertilization, respectively.

### Phthalate properties

DEHP (Oxoplast O 30) was generously provided by ZAK Spółka Akcyjna, Poland. Oxoplast is used as a plasticizer in PVC processing and in the paint and varnish industries. Oxoplast includes bis(2-ethylhexyl) phthalate at no less than 99.5% (m/m), phthalic acid at no more than 0.01% (m/m), and water at no more than 0.1% (m/m). Chemical impurities of DEHP include bis(2-ethylhexyl) terephthalate, 2-ethylhexyl benzoate and diisobutyl phthalate, dioctyl ether, and residual 2-ethylhexanol.

### Sample collection

All samples were collected into glass bottles or tubes to minimize accidental leakage or contamination of the samples with phthalates or other compounds. Blood was taken from the coccygeal vein or artery using BD Vacutainer-K_2_EDTA blood-collection tubes (Becton, Dickinson and company, Franklin Lakes, NJ). Samples were centrifuged (1,150 × *g* for 20 min), pooled per group and day, and stored in 30-ml glass tubes (Thermo Fisher Scientific, Waltham, MA) at -20°C for further analysis. Urine samples were collected directly from the bladder using an inserted tube. Samples were pooled per group and day and stored in 1-liter glass bottles (Thermo Fisher Scientific) at -20°C for further analysis. Milk samples for control cows were taken during routine milking. DEHP-treated cows were milked once a day with a portable milking machine. From each group and for each day, samples were pooled and stored in 0.5-liter glass bottles (Thermo Fisher Scientific) at -20°C until analysis.

### Ovarian scanning and FF aspiration

Ovarian scanning was performed throughout an entire estrous cycle (Fig 1A) with an ultrasound device (ALOKA-SSD-900, Tokyo, Japan) connected to a 7.5-MHz ultrasound linear probe. The position, diameter and number of follicles and corpus luteum were recorded. Follicles were categorized as small (3–5 mm), medium (6–9 mm) or large (>10 mm) according to their diameters. Follicles with diameter >25 mm were defined as potentially capable of developing into a persistent follicle or ovarian follicular cyst. The volume of the corpus luteum (in mm^3^) was calculated by its radius (R in mm) based on the scanned diameter, as 4 × π × R^3^/3.

Synchronized cows were treated with 2.5 ml PGF_2α_ to induce luteal regression, and the FF from the preovulatory follicle was aspirated 30 h later, as previously described [41]. Briefly, cows were sedated with 2 ml xylazine hydrochloride administered i.m. and caudal epidural anesthesia was induced with 5 ml lidocaine. Aspiration was performed with an ultrasound scanner (Pie Medical, Maastricht, Netherlands) equipped with a 7.5- MHz vaginal transducer and 19-gauge needle connected to a sterile syringe. The aspirated FF was centrifuged (250 x *g* for 10 min) and kept at -20°C until estradiol and phthalate metabolite analysis.

### Analysis of DEHP metabolites in the blood, urine and milk

#### Sample preparation

Plasma samples were centrifuged at 9.5 x *g*. Thereafter, both urine and plasma samples (1000 μl each) were spiked with a mixture of isotopically labeled standards (10 μl, 15 ng of each per sample), diluted with 1 ml sodium acetate solution (100 mM, pH 4.6 ml) and vortexed. All samples were cleaned on SPE cartridges (Strata-X, 60 mg, Phenomenex) using the following protocol: (1) conditioning with 1 ml methanol; (2) equilibration with 1 ml methanol/water (5:95); (3) loading the sample; (4) washing with 2 ml methanol/water (5:95); (5) drying was performed under full vacuum for 15 min; (6) elution with 1.3 ml acetonitrile.

Milk samples (2000 μl) were spiked with a mixture of isotopically labeled standards (20 μl, 30 ng of each per sample) diluted with 1 ml sodium acetate solution (100 mM, pH 4.6 ml) and vortexed. The samples were extracted with ethyl acetate (3 ml), vortexed and the emulsion was separated into two phases by centrifugation at 9.5 x *g* for 15 min. The organic phase layer was separated and ethyl acetate was evaporated under a nitrogen stream. Fatty residue was extracted with 200 μl acetonitrile. The acetonitrile phase was analyzed by LC–MS/MS.

#### LC–MS/MS analysis

Chromatographic analysis was performed using an Agilent 1200 Rapid Resolution LC system (Agilent Technologies Inc., Santa Clara, CA) consisting of vacuum micro degasser G1379B, binary pump G1312B, autosampler G1367C and thermal column compartment G1316B. HPLC separations were carried out using an Acclaim RSLC C18 HPLC column (2.1 × 150 mm, particle size 2.2 μm, Dionex) and gradient elution (solvent A—water with 0.1% acetic acid and solvent B—acetonitrile with 0.05% acetic acid). Other parameters were as follows: flow rate– 0.3 ml/min; temperature of column– 40°C; injection volume– 5 μl. The liquid chromatograph was coupled with an Agilent 6410B triple quad mass selective detector equipped with an electrospray ionization ion source. The mass spectrometer was operated in negative ionization mode, and ion source parameters were as follows: capillary voltage 3500 V, drying gas (nitrogen) temperature and flow 320°C and 10 l/min, respectively, nebulizer pressure 35 psi, and nitrogen (99.999%) was used as the collision gas. The LC–MS system was controlled and data were analyzed by MassHunter software (Agilent Technologies Inc.).

### Analysis of phthalate metabolites in the FF

Phthalate metabolites were analyzed as previously described [16]. Briefly, for each experimental group and for each aspiration day, FF aspirated from preovulatory follicles was pooled, frozen and sent to AXYS Analytical Services Ltd. (Sidney, British Columbia) for phthalate-metabolite analysis. Samples were extracted in accordance with AXYS method MLA-059 "Analytical Procedure for the Analysis of Phthalate Ester Metabolites in Urine” based on a previously described method [42]. Target compounds were analyzed using a Waters 2695 HPLC system (Milford, MA) coupled to ESI–MS/MS triple quad MS, Micromass Quattro Ultima (Waters Inc.) running MassLynx 4.1 software. Final concentrations of the metabolites: mono-methyl phthalate (MMP), mono-ethyl phthalate (MEP), mono-n-butyl phthalate (MBP), mono-benzyl phthalate (MBzP), MEHP, 5oxo-MEHP and 5OH-MEHP were determined by isotope dilution/internal standard quantification procedure. Details of the internal standards used to quantify each target are given in Table 1. For all target compounds, linear equations were determined from a 7-point calibration series for phthalate metabolites with 1/X weighting fit. Reported limits were the greater of the lowest calibration standard concentration equivalent or the sample detection limit (SDL). SDLs were determined by converting the area equivalents for three times the highest chromatographic noise to concentration. The concentration of 4-methylumbelliferone was used to monitor the efficiency of deconjugation by β-glucuronidase.

**Table 1:** Analytes, ions, and quantification references.

Target analyte	Parent ion mass	Daughter ion mass	Quantified against
Mono-methyl phthalate (MMP)	179	107	^13^C_4_-Mono-methyl phthalate
Mono-ethyl phthalate (MEP)	193	121	^13^C_4_-Mono-ethyl phthalate
Mono-butyl phthalate (MBP) (n-butyl/iso-butyl)	221	77	^13^C_4_-Mono-n-butyl phthalate
Mono-(3-carboxypropyl) phthalate (MCPP)	251	103	^13^C_4_-Mono-(3-carboxypropyl) phthalate
Mono-benzyl phthalate (MBzP)	255	183	^13^C_4_-Mono-benzyl phthalate
Mono-cyclohexyl phthalate (MCHP)	247	97	^13^C_4_-Mono-cyclohexyl phthalate
Mono-2-ethylhexyl phthalate (MEHP)	277	134	^13^C_4_-Mono-2-ethylhexyl phthalate
Mono-(2-ethyl-5-oxohexyl) phthalate [DEHP Metabolite VI] (MEOHP)	291	121	^13^C_4_-Mono-(2-ethyl-5-oxohexyl) phthalate
Mono-(2-ethyl-5-hydroxyhexyl) phthalate [DEHP Metabolite IX] (MEHHP)	293	121	^13^C_4_-Mono-(2-ethyl-5-hydroxyhexyl) phthalate
Mono-iso-nonyl phthalate (MiNP)	291	247	^13^C_4_-Mono-iso-nonyl phthalate
***Surrogate Standard***			
^13^C_4_-Mono-methyl phthalate	183	109	^13^C_4_-Mono-n-octyl phthalate
^13^C_4_-Mono-ethyl phthalate	197	124	^13^C_4_-Mono-n-octyl phthalate
^13^C_4_-Mono-n-butyl phthalate	225	79	^13^C_4_-Mono-n-octyl phthalate
^13^C_4_-Mono-(3-carboxypropyl) phthalate	255	103	^13^C_4_-Mono-n-octyl phthalate
^13^C_4_-Mono-benzyl phthalate	259	186	^13^C_4_-Mono-n-octyl phthalate
^13^C_4_-Mono-cyclohexyl phthalate	251	97	^13^C_4_-Mono-n-octyl phthalate
^13^C_4_-Mono-2-ethylhexyl phthalate	281	137	^13^C_4_-Mono-n-octyl phthalate
^13^C_4_-Mono-(2-ethyl-5-oxohexyl) phthalate	295	124	^13^C_4_-Mono-n-octyl phthalate
^13^C_4_-Mono-(2-ethyl-5-hydroxyhexyl) phthalate	297	124	^13^C_4_-Mono-n-octyl phthalate
^13^C_4_-Mono-iso-nonyl phthalate	295	250	^13^C_4_-Mono-n-octyl phthalate
^13^C_4_-4-methylumbelliferone	179	150	^13^C_4_-Mono-n-octyl phthalate
***Recovery Standard***			
^13^C_4_-Mono-n-octyl phthalate	281	127	External standard
***Deconjugation Recovery Standard***			
4-methylumbelliferone	175	147	13C4-4-methylumbelliferone

### Hormone concentrations

Estradiol concentration in the FF was analyzed using the solid-phase RIA kit (Siemens Medical Solutions Diagnostics, Los Angeles, CA) as previously described [43]. Assay sensitivity was 2.5 pg/ml and the intra- and interassay coefficients of variation were 6.9 and 5.5%, respectively.

Plasma progesterone concentration was analyzed by solid-phase RIA kit against a standard curve prepared from ovariectomized cow plasma [43]. The minimum detected amount was 0.2 ng/ml and the intra- and interassay coefficients of variation were 8.6 and 9.9%, respectively.

### In-vitro procedure

#### Chemicals and media

All chemicals, unless otherwise stated, were purchased from Sigma-Aldrich Co. (St. Louis, MO). The culture media Hepes–Tyrode's lactate (TL), sperm–TL (SP–TL) and in-vitro fertilization–TL (IVF–TL) were prepared in our laboratory as previously described [44,45]. Standard oocyte maturation medium (OMM) was made up of TCM-199 and Earle's salts supplemented with 10% (v/v) heat-inactivated fetal calf serum (Promega, Madison, WI), 0.2 mM sodium pyruvate, 50 μg/μl gentamicin, 2.2 g/l sodium bicarbonate, 2 μg/ml 17-_ß_ estradiol and 1.32 μg/ml FSH (Folltropin-V; Bioniche Animal Health, Belleville, Ontario, Canada). Potassium simplex optimized medium (KSOM) contained 95 mM NaCl, 2.5 mM KCl, 0.35 mM KH_2_PO_4_, 0.2 mM MgSO_4_·7H_2_O, 0.8% (v/v) sodium lactate, 0.2 mM sodium pyruvate, 0.2 mM D(+)-glucose, 25 mM NaHCO_3_, 0.01 mM phenol red, 1 mM L-glutamine, and 0.01 mM EDTA supplemented with 1.7 mM CaCl_2_·2H_2_O, 0.1 mg/ml polyvinyl alcohol, 100 U/ml penicillin-G, and 0.1 mg/ml streptomycin, 10 μl/ml essential amino acids and 5 μl/ml nonessential amino acids (Life Technologies, Carlsbad, CA).

#### In-vitro embryo production

Oocytes were matured in OMM (system control) and in FF aspirated from control cows (FF-control) or from DEHP-treated cows (FF-DEHP), as previously established in our laboratory [46]. Briefly, ovaries were obtained from a local abattoir and transported to the laboratory within 60 to 90 min in physiological saline solution (0.9% w/v NaCl at 38.5°C with 50 μg/ml penicillin–streptomycin). Cumulus oocyte complexes (COCs) were aspirated from 3- to 8-mm follicles with an 18-gauge needle attached to a 10-ml syringe. COCs were collected into Hepes–TL supplemented with 0.3% (w/v) bovine serum albumin (BSA), 0.2 mM sodium pyruvate and 0.75 mg/ml gentamicin (Hepes–TALP) at 38.5°C. At the end of the collection, COCs (n = 490–620 per group; seven replicates) with at least three layers of cumulus surrounding a homogeneous cytoplasm were selected for in-vitro maturation. Groups of 10 randomly selected COCs were matured in 50-μl droplets of FF-control or FF-DEHP supplemented with 1.32 μg/ml FSH overlaid with mineral oil at 38.5°C for 22 h in humidified air with 5% CO_2_. A subgroup of COCs was matured in standard OMM to validate the FF-culturing model.

At the end of maturation, COCs were washed three times in Hepes–TALP and transferred in groups of 30 oocytes to four-well plates containing 600 μl IVF–TALP and 25 μl PHE (0.5 mM penicillamine, 0.25 mM hypotaurine, and 25 μM epinephrine in 0.9% NaCl) per well. For IVF, COCs were co-incubated with Percoll-purified spermatozoa (~1 × 10^6^; ‘Sion’, Hafetz-Haim, Israel) for 18 h at 38.5°C in a humidified atmosphere with 5% CO_2_.

After fertilization, putative zygotes were denuded of cumulus cells by gentle vortexing in Hepes–TALP containing 1000 U/ml hyaluronidase, and placed in groups of 10 in 25-μl droplets of KSOM. All embryo droplets were overlaid with mineral oil and cultured for 7 days at 38.5°C in an atmosphere of humidified air with 5% CO_2_, 5% O_2_. Oocyte developmental competence was evaluated by the proportion of oocytes that cleaved into 2- to 4-cell-stage embryos 42–44 h postfertilization, and the proportion of embryos developed to blastocysts 7 days postfertilization.

### Nuclear and cytoplasmic maturation

#### Cumulus cell expansion

Oocytes were matured in OMM, FF-control or FF-DEHP. At the end of maturation, COCs (n = 45–100 per group; eight repetitions) were evaluated for cumulus cell expansion under a stereomicroscope (Nikon, Tokyo, Japan) and classified as having compact or fully expanded cumulus-cell layers.

#### Nuclear meiotic status

At the end of maturation, COCs (n = 20–36 oocyte/group; five repetitions) matured in OMM, FF-control or FF-DEHP were denuded of cumulus cells and washed three times in Hepes–TALP. The meiotic status of each oocyte was determined using the cell-permeant DNA dye DAPI. Oocytes were fixed in 2% (v/v) paraformaldehyde (Electron Microscopy Sciences, Hatfield, PA) in Dulbecco's phosphate buffered saline (PBS; Promega) for 15 min at room temperature and stored in PBS supplemented with 1 mg/ml polyvinylpyrrolidone (PBS–PVP) at 4°C until staining. Before staining, oocytes were washed three times in PBS–PVP and stained with 10 μg/ml DAPI in PBS–PVP for 15 min at room temperature. Nuclear status was examined under a Nikon inverted fluorescence microscope using Nis Elements software (Nikon). Oocytes were classified according to their nuclear stage into immature (germinal vesicle, GV; germinal vesicle breakdown, GVBD; metaphase I, MI; anaphase I, AI, and telophase I, TI) or mature (MII; i.e., metaphase II plate with an extruded first polar body).

#### Cortical granule distribution

At the end of maturation, a subgroup of COCs (n = 14–20 oocyte/group; three repetitions) matured in OMM, FF-control or FF-DEHP was stained with FITC–PNA to evaluate CG distribution. CG staining was performed as previously described [46]. Briefly, denuded oocytes were incubated in PBS–PVP supplemented with 0.1% (v/v) Triton X-100 (i.e., permeabilization solution) for 5 min at 39°C in an oven. Then oocytes were fixed in 2% paraformaldehyde (Electron Microscopy Sciences) and stored in PBS–PVP at 4°C. Oocytes were washed three times in PBS–PVP and placed in blocking solution [PBS supplemented with 1 mg/ml PVP, 0.1% v/v Triton X-100, 2% v/v normal goat serum, 0.1 M glycine, 1% w/v powdered skim milk (Becton, Dickinson and Company, Franklin Lakes, NJ) and 0.5% w/v BSA at pH 7.4] for 1 h at 39°C in the oven. After three washes in PBS–PVP, oocytes were stained with 100 μg/ml FITC–PNA in PBS–PVP for 30 min at 39°C in the oven. All oocytes were counterstained with 10 μg/ml DAPI and the pattern of CGs was analyzed under an inverted fluorescence microscope using Nis Elements software, and classified into the distributional pattern previously defined by Izadyar et al. [47]: type I–CG aggregates distributed throughout the cytoplasm, representing cytoplasmically immature oocytes; type II–CGs localized mainly in the cortical cytoplasm and distributed as individual particles as well as small aggregates, representing partially cytoplasmically mature oocytes; type III–CGs more or less evenly dispersed in the cortical cytoplasm lining the oolemma, representing fully cytoplasmically mature oocytes.

### Statistical analysis

Data were analyzed using JMP-7 software (SAS Institute Inc., 2004, Cary, NC). Means of the DEHP-metabolite concentrations in all fractions (plasma, urine and milk) before (day 0), during (days 2 and 4) and after (days 11, 19 and 24) exposure to DEHP were analyzed by one-way ANOVA followed by Student's *t* test. Data of follicle size, growth rate, corpus luteum volume, progesterone concentration and mean estradiol concentration were analyzed by one-way ANOVA followed by Student's *t* test. Data of the proportion of oocytes in different meiotic stages, cleavage rate into 2- to 4-cell-stage embryos and blastocyst-formation rate were arcsine-transformed before being subjected to one-way ANOVA followed by Tukey–Kramer test. Data are presented as mean ± SEM. Overall comparison of treatments for incidence data was performed by Pearson’s chi-square test. Variables were: follicular pathology, nuclear meiotic status, cumulus cell expansion and CG type. Pairs of treatments were also compared by chi-square test followed by Fisher's exact test. For all analyses, *P* < 0.05 was considered significant; *P-*values between 0.05 and 0.1 were also reported as trends that might be real and worthy of note.

## Results

### Phthalate-metabolite levels in plasma, milk and urine fractions

**Fig 2:**
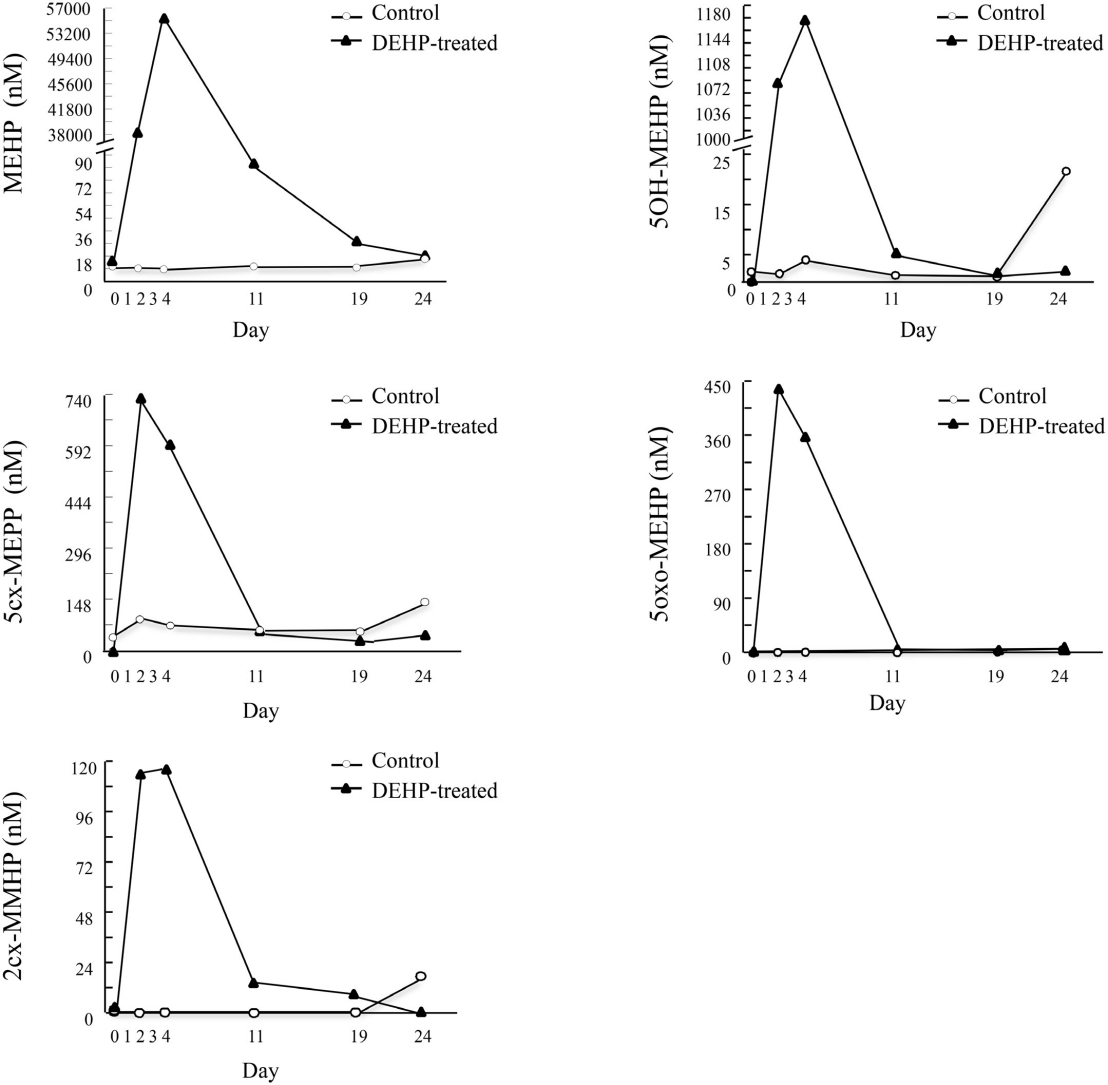
DEHP-metabolite concentrations in the plasma before, during and after DEHP exposure. Cows were administered with water (control) or 100 mg/kg DEHP per day on days 1–3 of the experiment (DEHP-treated). Blood samples were taken before (day 0), during (days 2 and 4) and after (days 11, 19 and 24) DEHP exposure. Samples were analyzed for concentrations of DEHP metabolites mono(2-ethylhexyl) phthalate (MEHP), mono(2-ethyl-5-hydroxyhexyl) phthalate (5OH-MEHP), mono(2-ethyl-5-oxohexyl) phthalate (5oxo-MEHP), mono(2-ethyl-5-carboxypentyl) phthalate (5cx-MEPP) and mono[2-(carboxymethyl)hexyl] phthalate (2cx-MMHP) by LC–MS/MS.

On day 0 of the experiment (i.e., before DEHP exposure), the average levels of all examined DEHP metabolites were relatively low in all three fractions (plasma, milk and urine). In the control group, DEHP-metabolite levels remained low in all collected fractions throughout the experiment and did not differ from levels detected on day 0 (Table 2). In the DEHP-treated cows, DEHP-metabolite levels increased (*P* < 0.05) in all examined fractions during DEHP exposure (days 2 and 4), followed by rapid clearance throughout the subsequent days of the experiment (days 11, 19 and 24) to levels similar to those detected in the control group (Table 2). For instance, the highest MEHP level in the plasma reached up to 56.58 μM during DEHP exposure and decreased to 5.74 nM over subsequent days to a level similar to those detected before DEHP exposure (12.21 nM) and in the control group (15.5 ± 2.9 nM; Fig 2).

**Table 2:** Phthalate-metabolite concentrations (nM) in pooled plasma, milk and urine samples collected before, during and after DEHP exposure.

Sample		MEHP	5OH-MEHP	5oxo-MEHP	2cx-MMHP	5cx-MEPP
Plasma	*Before exposure	10.78±1.4^a^	1.02±1.02^a^	0.17±0.17^a^	0.0±0.0^a^	20.29±20.29^a^
	**Control	15.5±2.9^a^	6.12±4.04^a^	1.03±0.6^a^	3.64±3.64^a^	84.74±16.84^a^
	*** During exposure	47690±8890^b^	1118.4±44.9^b^	398.63±39.73^b^	115.91±0.97^b^	665.97±67.79^b^
	**** After exposure	29.6±14.4^a^	1.9±1.07^a^	0.55±0.38^a^	5.39±3.39^a^	25.84±11.03^a^
Milk	Before exposure	8.44±2.34^a^	0.34±0.34^a^	0.0±0.0^a^	0.0±0.0^a^	19.64±0.49^a^
	Control	36.46±29.58^a^	2.72±1.48^a^	0.59±0.35^a^	0.0±0.0^a^	39.69±16.22^a^
	During exposure	415.5±197.1^b^	64.63±28.23^b^	28.08±12.67^b^	47.89±4.06^b^	480.03±6.98^b^
	After exposure	18.49±6.28^a^	0.85±0.85^a^	0.51±0.51^a^	0.0±0.0^a^	16.88±6.49^a^
Urine	Before exposure	53.0±19.0^a^	36.0±27.0^a^	6.5±4.5^a^	49.4±49.4^a^	153.0±120.0^a^
	Control	61.0±23.0^a^	49.0±15.0^a^	11.6±4.6^a^	0.0±0.0^a^	67.0±14.0^a^
	During exposure	243315±73235^b^	128265±26020^b^	40222.6±7551.4^b^	12243.5±3477.3^b^	124188±33604^b^
	After exposure	871±715^a^	871.0±548.0^a^	232.9±156.5^a^	213.3±84.1^a^	811±407^a^

*Before exposure = samples collected from all cows on day 0 of the experiment.

**Control = samples collected from control cows on days 2, 4, 11, 19 and 24 of the experiment.

***During exposure = samples collected from DEHP-treated cows on days 2 and 4 of the experiment.

****After exposure = samples collected from DEHP-treated cows on days 11, 19 and 24 of the experiment.

Data presented as mean ± SEM. Different superscript letters indicate significant difference at *P* < 0.05 within columns, for each sample and metabolite separately.

### Effect of phthalates on ovarian function

#### Follicular development

**Fig 3:**
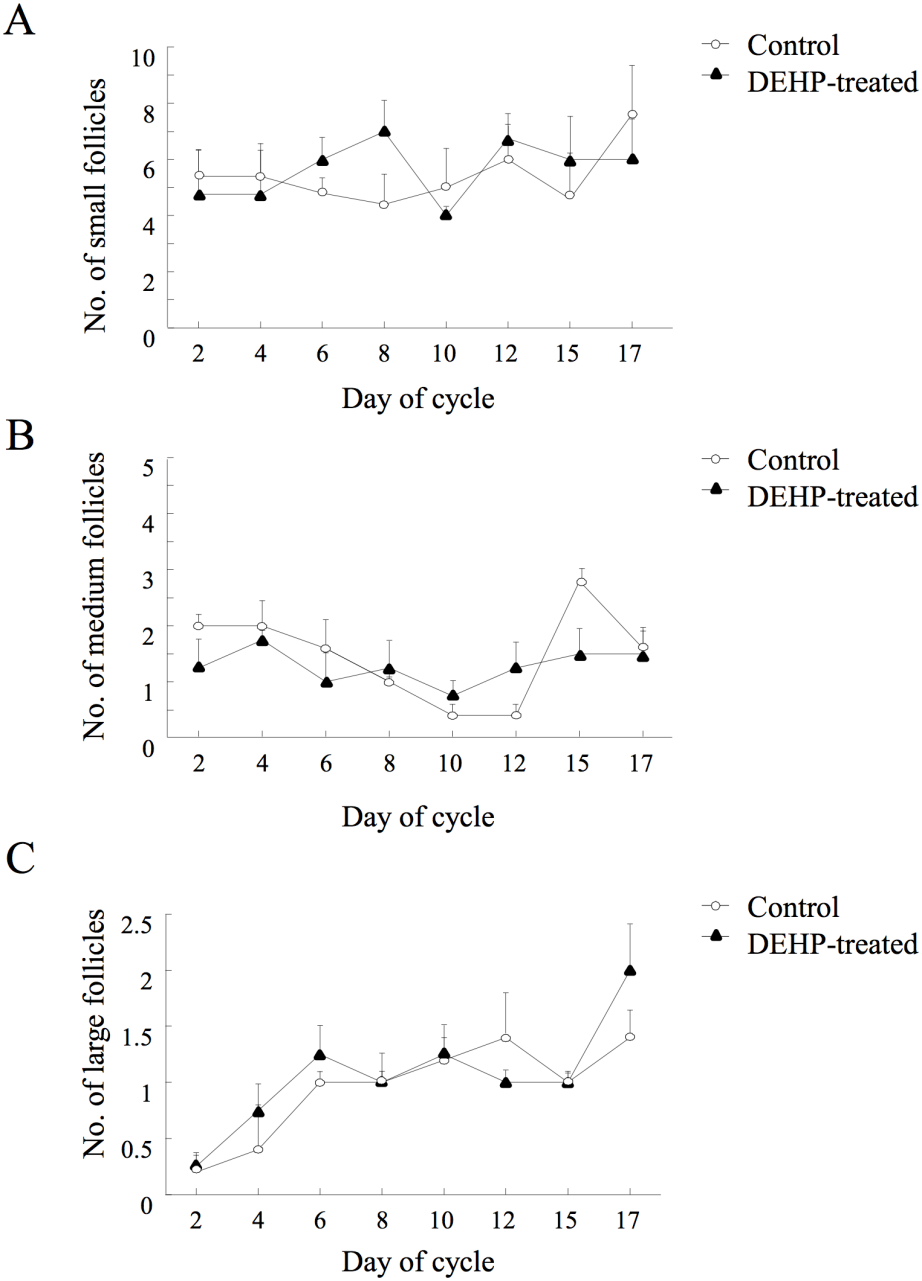
Effects of DEHP on follicular development. Control and DEHP-treated cows were synchronized and ovarian follicular dynamics was monitored by linear ultrasonographic scanner throughout an entire cycle. The positon and number of follicles were recorded. The numbers of (A) small (3–5 mm in diameter), (B) medium (6–9 mm in diameter), and (C) large (>10 mm) follicles are presented as means ± SEM.

The number of small-size follicles did not differ between the control and DEHP-treated groups through the examined cycle (Fig 3A). The number of medium-size follicles did not differ between the control and DEHP-treated groups during the examined cycle, but was numerically lower on day 15 in the DEHP-treated group relative to the control (Fig 3B). The number of large follicles did not differ between groups but was numerically higher on day 17 of the cycle in the DEHP-treated group relative to the control (Fig 3C).

**Fig 4:**
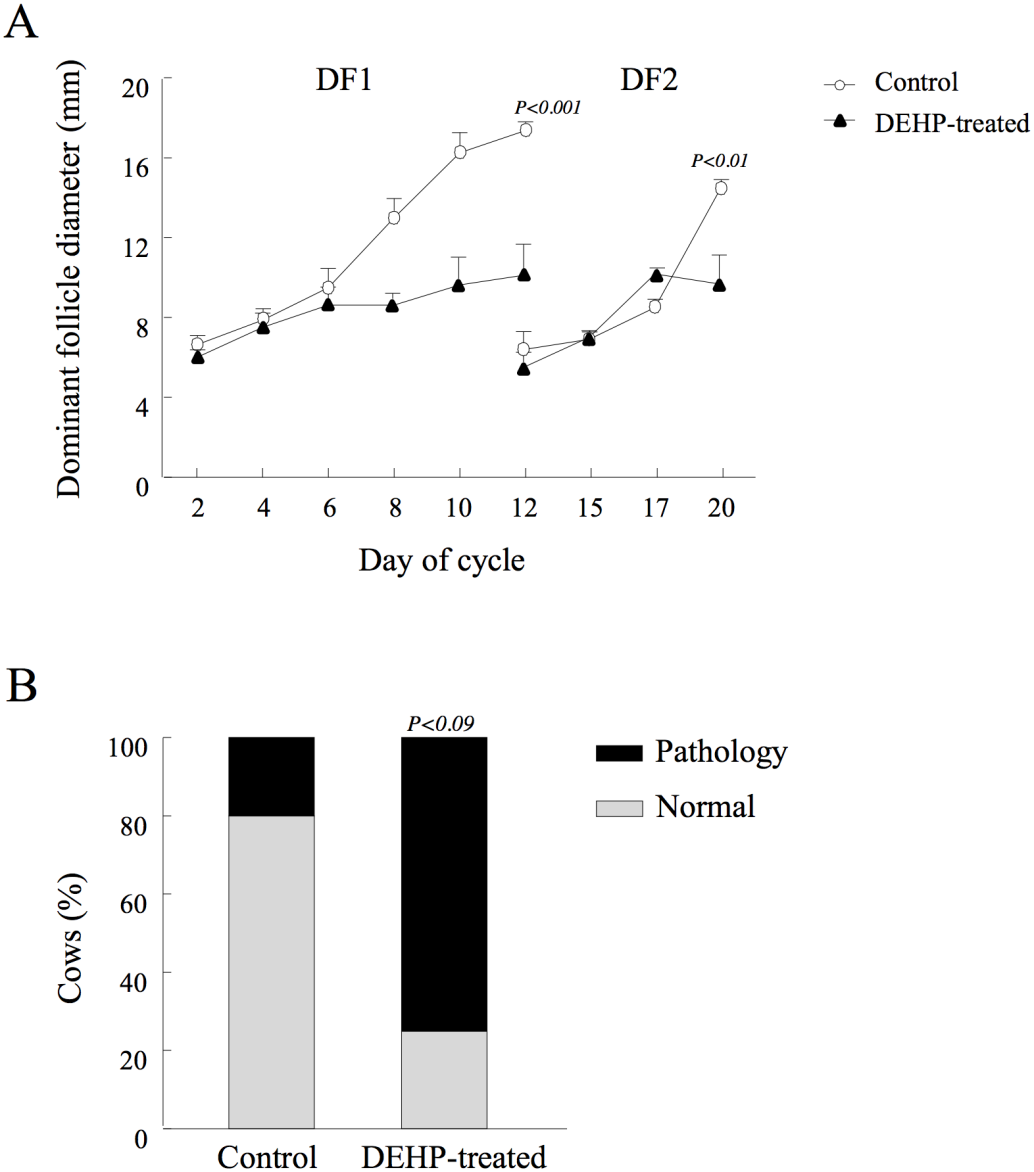
Effects of DEHP on diameter of first- and second-wave dominant follicles. Control and DEHP-treated cows were synchronized and ovarian follicular dynamics was monitored by linear ultrasonographic scanner throughout an entire cycle. The positon and diameter of the dominant follicles were recorded. (A) Mean diameters of first-wave dominant follicle (DF1) and second-wave dominant follicle (DF2). Data are presented as mean ± SEM; *P*-value indicates for treatment effect within experimental groups in each examined day. (B) Development of follicular pathologies, i.e., follicles with diameter >25 mm and defined as able to develop to a persistent follicle or ovarian follicular cyst. Presented are the proportions of control and DEHP-treated cows with follicular pathologies, calculated out of total cows for each experimental group. *P*-value indicates for treatment effect within experimental groups.

Phthalates impaired the pattern of follicular development, with a prominent effect on first- and second-wave dominant follicles (*P <* 0.001; Fig 4A). In particular, the growth rate of the first-wave dominant follicle was higher (*P* < 0.02) in the control than in the DEHP-treated group (0.88 ± 0.15 vs. 0.42 ± 0.18 mm/day, respectively); the maximal diameter on day 12 of the cycle was larger in the control than in the DEHP-treated group (*P* < 0.001; Fig 4A). Similarly, the growth rate of the second-wave dominant follicle was higher in the control group than in the DEHP-treated group (1.08 ± 0.06 vs. 0.56 ± 0.13 mm/day, respectively, *P* < 0.006) with a bigger diameter at the preovulatory stage (day 20 of the cycle) in the control than in the DEHP-treated group (*P <* 0.01; Fig 4A).

The proportion of dominant follicles that developed into follicular pathologies (diameter >25 mm) tended to be higher in the treated vs. control group (75 vs. 20%, respectively, *P* < 0.09; Fig 4B).

#### Corpus luteum development and progesterone concentration

**Fig 5:**
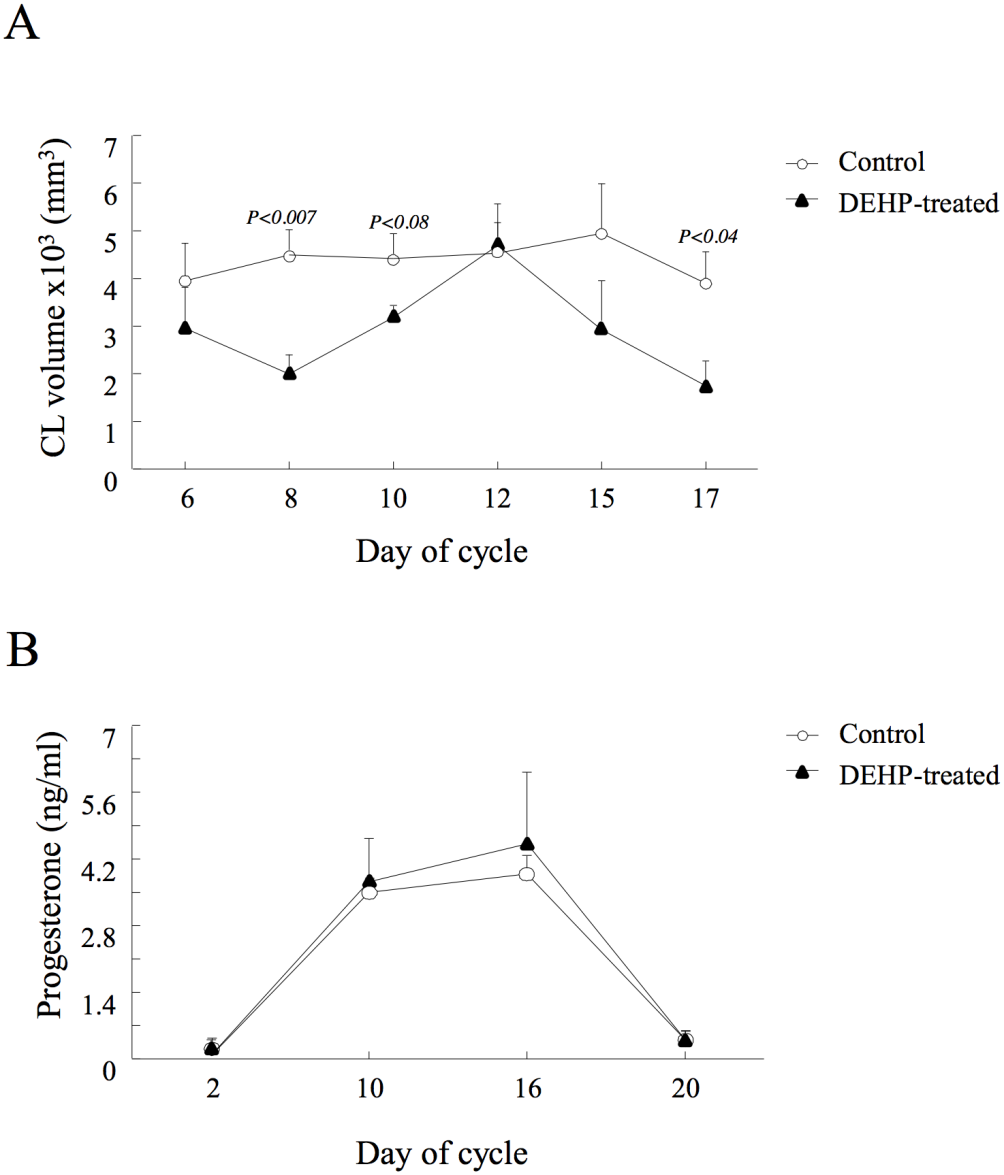
Effect of DEHP on corpus luteum (CL) size and function. Control and DEHP-treated cows were synchronized and ovarian follicular dynamics was monitored by linear ultrasonographic scanner throughout an entire cycle. (A) CL volume was calculated by its radius (R in mm), based on the scanned diameter, as 4 × π × R^3^/3, recorded on days 6–17 of the cycle. Data are presented as means ± SEM; *P-*value indicates treatment effect within experimental groups on each examined day. (B) Plasma progesterone concentrations during days 2–20 of the cycle. Data presented as means ± SEM; *P*-value indicates treatment effect within experimental groups on each examined day.

The pattern of growth and regression of the corpus luteum was impaired by the DEHP treatment (Fig 5A), as reflected by a slower developmental rate (days 6–12 of the cycle), with a significantly lower (*P* < 0.007) corpus luteum volume on day 8 (1.99 x 10^3^ ± 0.34 vs. 4.49 x 10^3^ ± 0.53 mm^3^) and a tendency (*P* < 0.08) to be lower on day 10 (3.18 x 10^3^ ± 0.17 vs. 4.42 x 10^3^ ± 0.52 mm^3^) in the DEHP-treated group vs. control, respectively. The maximal volume did not differ between groups on day 12 of the cycle. Earlier and faster regression of the corpus luteum was obtained in the DEHP-treated group, reflected by a significantly lower (*P <* 0.04) corpus luteum volume on day 17 relative to the control group (1.75 x 10^3^ ± 0.49 vs. 3.89 x 10^3^ ± 0.67 mm^3^, respectively; Fig 5A).

Overall, the average volume of the corpus luteum through the entire cycle was bigger (*P* < 0.05) in the control than in the DEHP-treated group (4.37 x 10^3^ ± 0.27 vs. 2.91 x 10^3^ ± 0.31 mm^3^, respectively). However, no functional difference was found between the control and DEHP-treated groups, reflected by similar pattern of plasma progesterone concentration (Fig 5B).

### Estradiol and phthalate content in FF

#### Estradiol concentration

**Fig 6:**
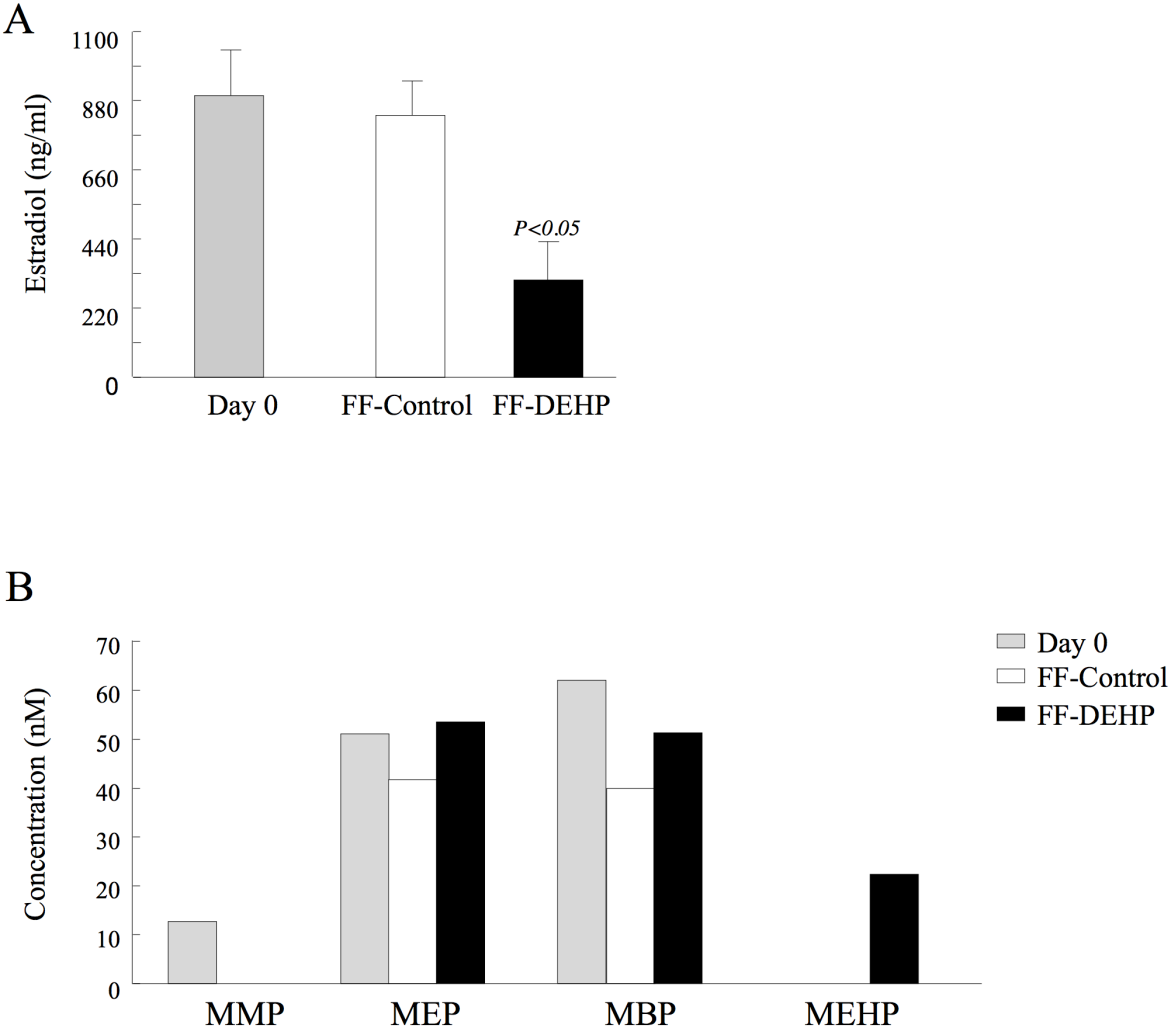
Estradiol and phthalate-metabolite concentrations in the follicular fluid (FF). Cows were tube-fed DEHP or water (control) on days 1–3 of the experiment. FFs of the preovulatory follicles were aspirated before (day 0) and after (day 29) DEHP treatment. Data presented as mean ± SEM; *P*-value indicates for treatment effect within experimental groups on each examined day. (B) Phthalate-metabolite concentrations in FF on day 0 and day 29 of the experiment. MMP, mono-methyl phthalate; MEP, mono-ethyl phthalate; MBP, mono-n-butyl phthalate; MEHP, mono(2-ethylhexyl) phthalate.

The average estradiol concentration in the FF aspirated from preovulatory follicles before DEHP administration (day 0; all cows) did not differ from that found in the control group following the treatment (Fig 6A). In contrast, estradiol concentration in the FF was lower (*P < 0*.*05*) in the DEHP-treated vs. control groups (361.61 ± 130.5 vs. 832.6 ± 109.7 ng/ml; Fig 6A).

#### Phthalate content

FF of the preovulatory follicles aspirated before DEHP administration did not contain DEHP metabolites. On the other hand, 26 days after treatment ended, 22.31 nM MEHP was found in the FF of the DEHP-treated, but not in the control group (Fig 6B). Other DEHP metabolites (5oxo-MEHP and 5OH-MEHP) were not detected in the FFs of either group.

FF aspirated before treatment contained other phthalates that did not originate from DEHP such as MMP, MEP and MBP (Fig 6B). The metabolites MEP and MBP were also detected 26 days after DEHP administration, but their concentration did not differ between groups.

### Effect of phthalates on oocyte competence

#### Oocyte maturation

**Fig 7:**
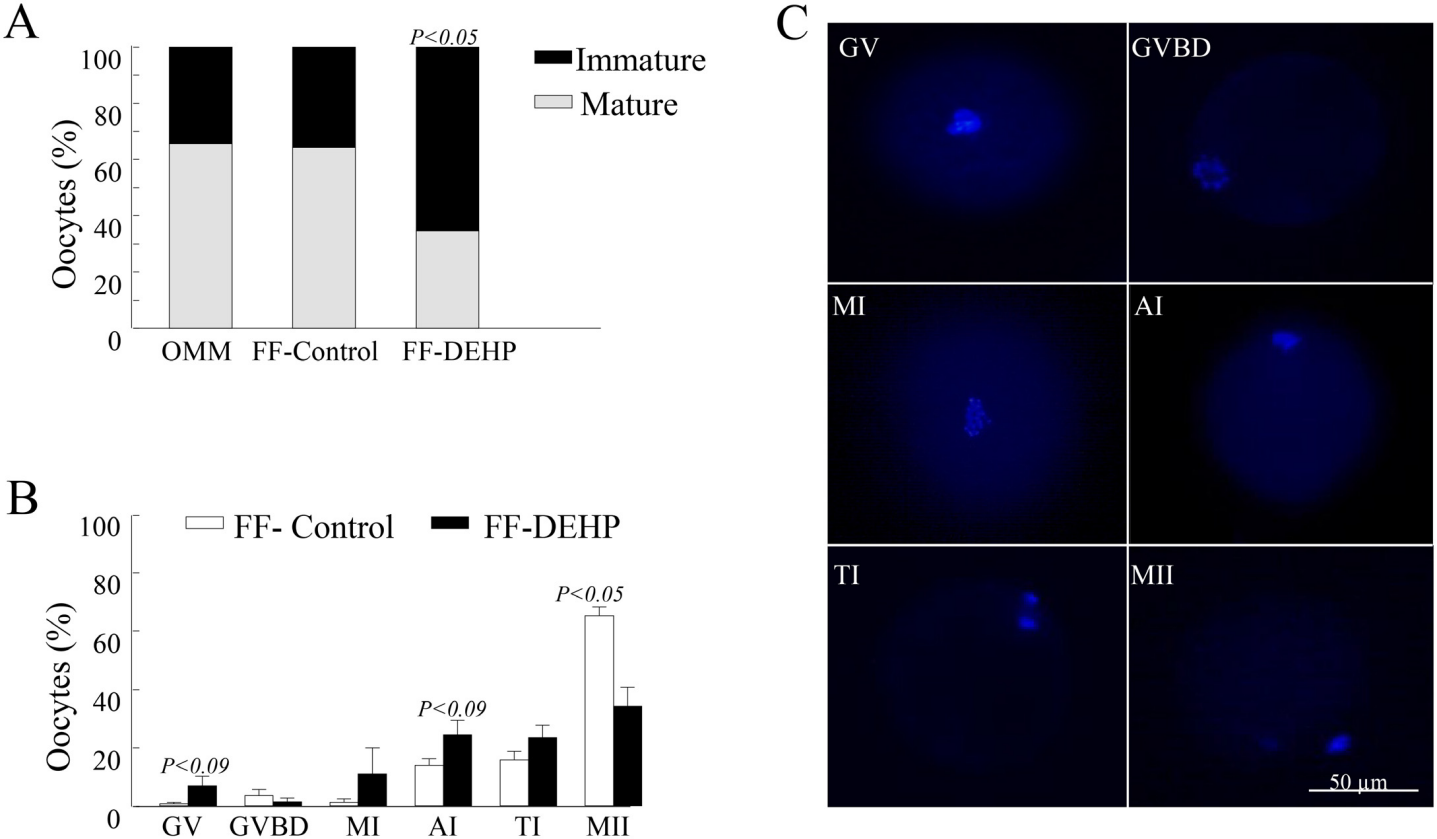
Nuclear maturation of oocytes cultured in follicular fluid (FF) aspirated from control (FF-control) and DEHP-treated (FF-DEHP) cows, or in oocyte maturation medium (OMM). At the end of maturation, oocytes were denuded and stained with DAPI to evaluate their meiotic status. (A) Proportion of oocytes at MII stage (i.e., mature) or at earlier meiotic stages (i.e., immature) calculated out of total oocytes; *P*-value indicates treatment effect within maturation stages between experimental groups. (B) Distribution of oocytes into meiotic stages. Data are presented as means ± SEM; *P*-value indicates treatment effect within meiotic stages between experimental groups: germinal vesicle (GV); GV breakdown (GVBD); metaphase I (MI); anaphase I (AI); telophase I (TI), and metaphase II (MII). (C) Representative images of oocytes at different meiotic stages:

The proportion of COCs with expanded cumulus cells at the end of 22 h maturation did not differ between COCs matured in standard OMM and those matured in FF-control (84.51 and 84.5%, respectively). Meiotic progress did not differ between groups since the proportion of mature (i.e., MII stage) and immature (i.e., GV, GVBD, MI, AI or TI stages) oocytes did not differ between the OMM and FF-control groups (64.29 and 35.71% vs. 65.67 and 34.33%, respectively; Fig 7A).

Cumulus cell expansion did not differ between the experimental groups, as reflected by the similar proportion of COCs with expanded cumulus cells in the FF-DEHP-treated and FF-control groups (81.2 and 84.5%, respectively). However, the proportion of MII-stage oocytes was lower (*P* < 0.0001) in oocytes cultured in FF-DEHP than in FF-control (34.67 and 65.67%, respectively; Fig 7A) and the proportion of GV- and AI-stage oocytes tended to be higher (*P* < 0.09) in FF-DEHP than in FF-control (Fig 7B and 7C).

**Fig 8:**
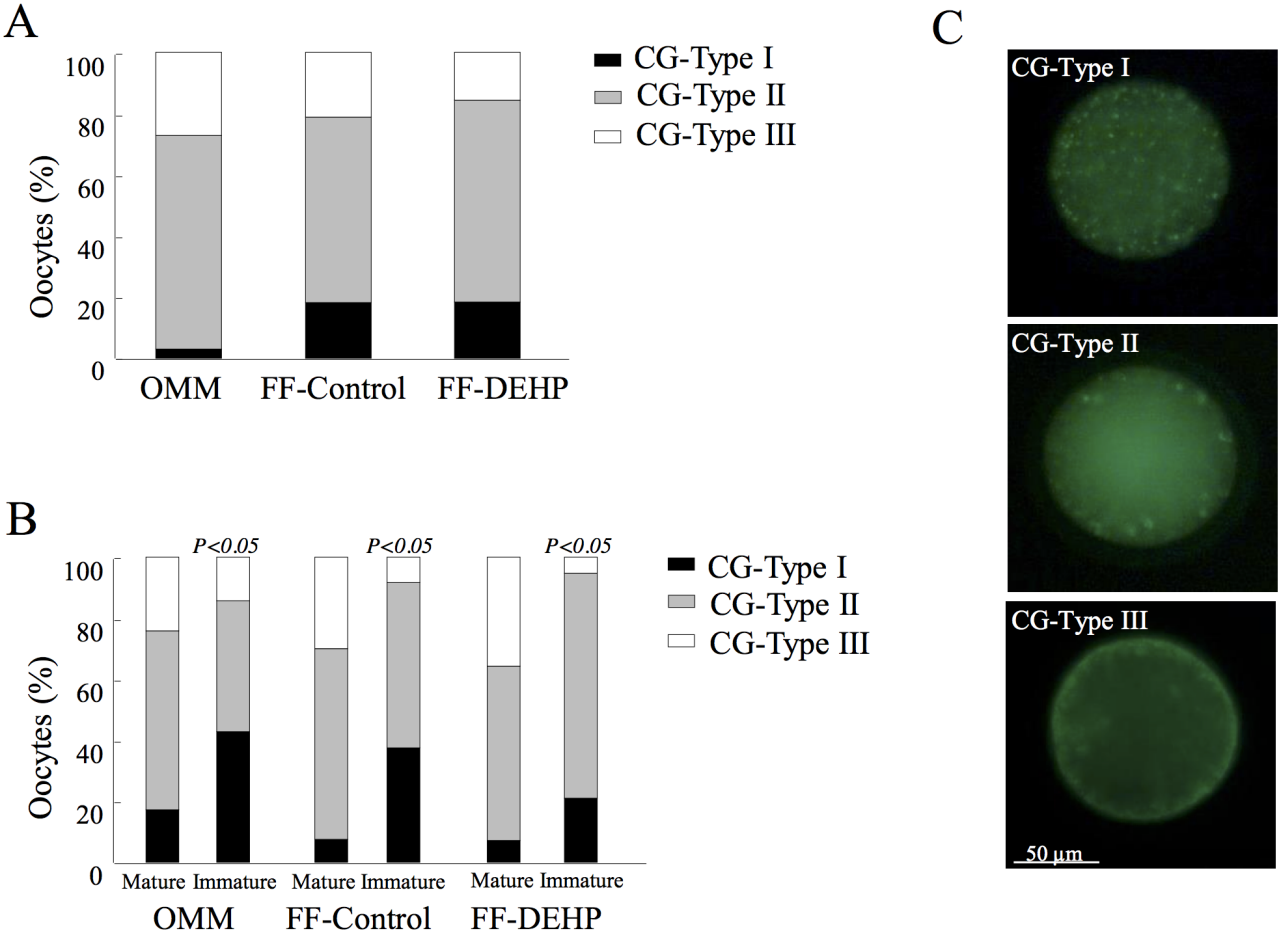
Cortical granule (CG) distribution in oocytes matured in follicular fluid (FF) aspirated from control (FF-control) and DEHP-treated (FF-DEHP) cows, or in oocyte maturation medium (OMM). At the end of maturation, oocytes were denuded, fixed and stained with FITC–PNA to evaluate the pattern of CG distribution and counterstained with DAPI. (A) Proportion of oocytes in each CG-distribution type, calculated out of total oocytes for each experimental group. (B) Distribution of CG types within mature and immature oocytes for each experimental group. *P*-values indicate different CG-distribution patterns within meiotic stages for each experimental group. (C) Representative images of CG-distribution patterns in oocytes: CG type I, characterized by CG aggregates, representing cytoplasmically immature oocytes; CG type II, characterized by CGs localized mainly in the cortical cytoplasm and distributed as individual particles as well as small aggregates, representing partially cytoplasmically mature oocytes; CG type III, characterized by CGs more or less evenly dispersed in the cortical cytoplasm lining the oolemma, representing fully cytoplasmically mature oocytes.

Oocyte distribution into CG type I–III did not differ between FF-control and OMM groups or FF-DEHP groups (*P* < 0.7; Fig 8A). Further analysis taking into account both meiotic status and CG type revealed differences (*P* < 0.05) in CG distribution between mature and immature oocytes for both FF-control and FF-DEHP groups (Fig 8B). In the FF-control group, the proportion of type I CGs was higher (*P* < 0.006) and that of type III CGs was lower (*P* < 0.04) in immature vs. mature oocytes. Similarly, in the FF-DEHP group, the proportion of type III CGs was lower (*P* < 0.02) in immature vs. mature oocytes (Fig 8B and 8C).

#### Oocyte developmental competence

**Fig 9:**
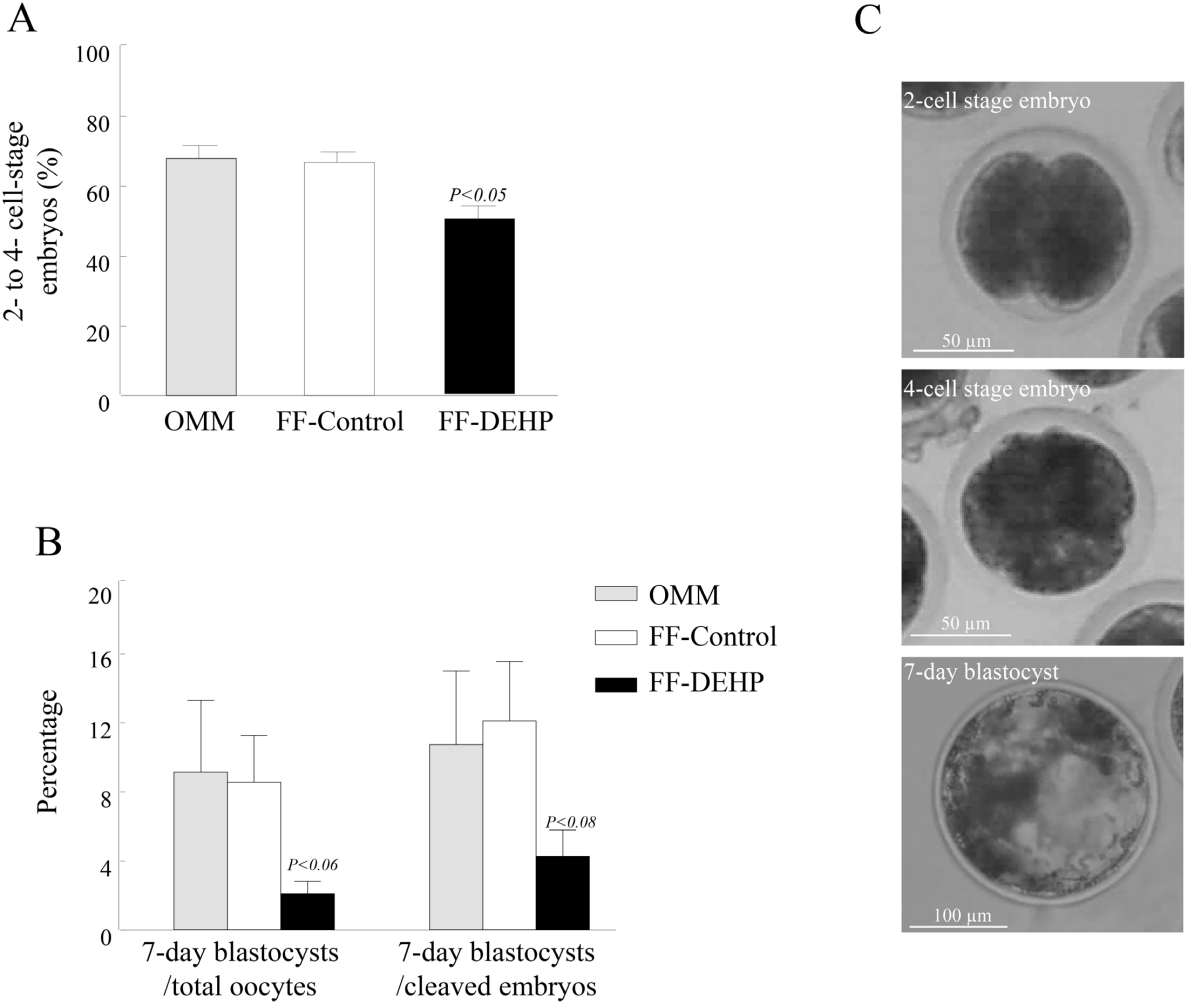
Developmental competence of bovine oocytes matured in follicular fluid (FF) aspirated from control (FF-control) and DEHP-treated (FF-DEHP) cows. (A) Proportion of oocytes cleaved to 2- to 4-cell-stage embryos 42–44 h postfertilization, and (B) proportion of embryos developed to the blastocyst stage on day 7 postfertilization, calculated from total oocytes or from cleaved embryos, respectively. Data are presented as means ± SEM; *P*-value indicates for treatment effect within embryonic stages between experimental groups. (C) Representative images of oocytes that were fertilized and cleaved into 2-cell-stage embryos, 4-cell-stage embryos, and further developed to 7-day blastocysts.

Oocyte developmental competence was similar between oocytes cultured in OMM and those cultured in FF-control, reflected by a similar proportion of oocytes fertilized and cleaved to 2- to 4-cell-stage embryos (67.8 ± 3.9 and 66.6 ± 3.1%, respectively, *P* < 0.8), and a similar proportion of blastocysts developed from cleaved embryos (10.6 ± 4.2 and 11.9 ± 3.4%, respectively; *P* < 0.8; Fig 9).

The developmental competence of oocytes cultured in FF-DEHP was impaired, reflected by a decreased proportion of oocytes cleaved to 2- to 4-cell-stage embryos (*P* < 0.04; Fig 9A) and further developed to the blastocyst stage (*P* < 0.08; Fig 8B and 8C).

## Discussion

To the best of our knowledge, this is the first description of the clearance of DEHP and its metabolites from the circulation, milk and urine of lactating cows. The study also explored the carryover effects of acute exposure to DEHP on ovarian function, including growth of the small, medium, dominant and preovulatory follicles, as well as the corpus luteum. Use of an ex-vivo model in which oocytes were matured in FF aspirated from control or DEHP-treated cows enabled exploring the deleterious effects of this compound on oocyte maturation and developmental competence.

Studies in rats and mice orally administered with DEHP have reported an LD_50_ of >20,000 mg/kg and >10,000 mg/kg, respectively with a toxic effect on the testes [48]. Similarly, exposure of pregnant rats to 405 mg/kg DEHP did not show maternal toxicity but impaired reproduction in both male and female offspring [34,49]; and doses >200 mg/kg have adverse effects on fetal development [50]. In the current study, cows were exposed to 100 mg/kg DEHP per day (i.e., in the safe range of toxicity), to mimic acute exposure; its carryover effect on ovarian function was examined a few days later, when DEHP-metabolite concentrations in the plasma had decreased to relatively low levels (0.55–29.6 nM). This finding is highly relevant because a similar DEHP concentration of 6 ppb or 6 μg/l (~15 nM) is considered safe for drinking water by the US Environmental Protection Agency [51].

Once they enter the body, diester phthalates are rapidly hydrolyzed into monoester forms [4,9]. These biomarkers have been found in the liver, kidney and testis of rats, marmosets and mice [52,53]; in the fat and muscle tissues of sheep [20], in bovine fat tissue [19]; and in the blood of humans [10], mice [54], rats and marmosets [52]. In addition, DEHP has been detected in female sheep plasma 29 days after injection [55]. In the current study, DEHP metabolites were not detected in any of the cows before DEHP administration, or in untreated control cows throughout the experimental period. A low concentration of DEHP metabolites was found in the milk samples of the treated cows 20 days after DEHP administration, exposing the nursing calf to the potential risk of DEHP metabolites via the milk, as has been previously suggested in lactating women [56]. In humans, the excretion of phthalate metabolites via the urine or feces is completed within 1 or 2 days [57]. However, in the current study, DEHP levels in the urine remained relatively high on day 20 after DEHP administration. Differences between studies might be due to the high dose used in the current study as well as to differences in metabolism and clearance rates between species. Given that phthalate metabolites in the urine are considered a biomarker for phthalate contamination, this point should be further examined.

One of the potential risks of DEHP is the passage of its monoester forms from the peripheral blood, across the placental barrier to the embryo. Radiolabeled DEHP [53,54] and MEHP [53] administered to pregnant mice during gestation passed into the developing fetus. Mice receiving 1 ml/kg MEHP orally on days 8–9 of gestation produced a lower number of live and healthy pups [58]. Since the blood-barrier threshold of the follicular basal lamina is 100 kDa [59], it is reasonable to assume that small molecules such as MEHP (278.34 Da) pass from the circulation into the FF. Since the FF is of plasma origin [60], it is reasonable to assume that DEHP metabolites in the plasma might also affect FF contents. In the current study, FF aspirated 26 days after DEHP administration was complete contained 22.31 nM MEHP, most likely a residual concentration left after entering the follicle and then being cleared to the circulation.

It should be noted that FF from both control and treated groups also contained some metabolites that were not of DEHP origin, such as MEP and MBP, presumably from an environmental source. As MEP and MBP concentrations did not differ between the groups, it is likely that MEHP, rather than the other metabolites, was responsible for impairing the maturation and developmental competence of the oocytes in the treated group. In support of this, phthalate-metabolite contents in FF aspirated from five women undergoing IVF treatment ranged between 21.45 and 51 nM [16], with an average of 33.62 nM, similar to the concentrations reported here. Nevertheless, a cumulative effect on the oocytes due to interactions between MEP, MBP and MEHP cannot be ruled out. Interestingly, in both humans [16] and the current bovine study, DEHP metabolites such as 5oxo-MEHP and 5OH-MEHP, which have longer half-lives and are considered DEHP biomarkers [9], were not detected in the aspirated FF.

As growth from an antral (0.5–1.0 mm in diameter) to preovulatory (15–18 mm) follicle takes about two estrous cycles [61], the findings of the current study suggest a deleterious carryover effect of DEHP administration on follicular development. This was expressed by low growth rate and small diameter of the dominant follicles in DEHP-treated cows. In rodents, DEHP impairs follicle growth at different stages of development. Injection of DEHP into both pubertal age and adult female mice altered follicular development by decreasing the number of primordial follicles and increasing the number of antral and secondary follicles [62]. Administration of DEHP to mice for 10 days impaired early folliculogenesis as expressed by a lower percentage of primordial and primary follicles [31]. In-vitro culture of mouse antral follicles with DEHP or MEHP caused a decrease in their growth rate [32,63]. Suppression of follicular development was detected in rat secondary follicles cultured in vitro with MEHP [64]. However, less is known about the effect of DEHP on dominant and preovulatory follicles in large domestic animals. Based on the ultrasonography records, DEHP administration did not have a significant effect on the number of small-, medium- or large-size follicles during the estrous cycle. However, a reduced number of medium-size follicles in DEHP-treated cows on day 15 of the estrous cycle might suggest alteration in follicular growth. Furthermore, the increased number of large follicles during the follicular phase indicated reduced dominance of the preovulatory follicle associated with reduced steroidogenic capacity (discussed below). In addition, the growth rate and the maximal diameter of the dominant and preovulatory follicles were much smaller in the DEHP-treated group. It should be noted, however, that diameter of itself is not a good indicator of follicular function [65].

Previous studies have reported a decline in serum estradiol concentration in mice and rats treated with DEHP [29,66–68]. In addition, ovaries obtained from rats orally exposed to DEHP showed impaired estradiol and testosterone levels [69]; prepubertal female rats exposed to DEHP via inhalation had increased estradiol levels in the serum [70]. While not yet completely clear, the reduced estradiol concentration in the FF aspirated from DEHP-treated cows might reflect a direct effect of either DEHP or its metabolite, MEHP, on follicular cells. Supporting this, in-vitro culture of mouse antral follicles with DEHP and MEHP reduced estradiol production in a dose-dependent manner [32]. MEHP has been found to reduce estradiol production in human granulosa lutein cells [71] and in rat granulosa cells cultured with this compound [29]. Moreover, a comparison of the effects of different monoester phthalates on estradiol production in rat granulosa cells revealed a prominent effect of MEHP [72]. In contrast, in-vitro culture of mouse follicles with MEHP did not affect estradiol production [73]. As DEHP itself has some intrinsic cellular activity [40], direct effects of DEHP and its metabolites on the hypothalamus–pituitary–ovarian (HPO) axis cannot be ruled out. Oral administration of DEHP to adult female rats has been found to disrupt the HPO axis, as reflected by an increased level of hypothalamic GnRH, in association with lower levels of FSH, LH, progesterone and estradiol in the serum [68]. DEHP administration to immature female rats reduced progesterone and estradiol levels in association with increased LH levels in the serum [67]. Inhalation of DEHP increased serum LH level in prepubertal female rats [70]. Taken together, the reduced steroidogenic capacity and potential alterations in the HPO axis might underlie the increase in ovarian pathologies (i.e., follicles with diameter >25 mm) in the treated cows. In fact, three out of four cows had ovarian pathologies following DEHP administration. These pathologies are likely to affect fertility, disrupt cyclicity and recruitment of new follicular waves, and impair ovulation of the preovulatory follicle [74].

DEHP administration in one cycle also impaired the formation and regression dynamics of the corpus luteum in the subsequent cycle, characterized by slow growth rate, as well as earlier and faster luteolysis. One might expect these structural alterations to be associated with reduced steroidogenesis in the corpus luteum. However, plasma progesterone concentration was normal and did not differ between control and DEHP-treated cows at any point in the estrous cycle. Similarly, exposure of adult sheep to DEHP results in a smaller corpus luteum, but with higher plasma progesterone levels [55]. In-vitro culture of rat secondary follicles with 100 μg/ml MEHP increased progesterone levels in the culture media [64]. Higher progesterone levels were also detected after culturing porcine COCs with 1 μM DEHP [75]. In contrast, studies in mice [76] and immature rats [67] reported that administration of DEHP reduces serum progesterone level. Differences between studies might be due to different experimental approaches (i.e., in vivo vs. in vitro), as well as different doses and routes of exposure, and different DEHP-kinetics patterns in the different animal models [21,24].

The passage of metabolites from the plasma into the FF might also affect the follicle-enclosed oocyte. The findings of the current study confirmed this assumption and revealed a carryover effect on the intrafollicular microenvironment associated with acute phthalate exposure. In particular, culturing oocytes in FF aspirated from DEHP-treated cows and containing 22.31 nM MEHP impaired the proportion of oocytes that resume meiosis and progress to the MII stage. This was associated with a reduced proportion of oocytes cleaving and developing to blastocysts. In previous studies, we reported that exposing oocytes to 50 μM MEHP during maturation impairs meiotic progression and cytoplasmic maturation and reduces oocyte developmental competence [37,38]. Another study in bovines reported a negative impact on nuclear maturation of oocytes cultured with 10 and 25 μM MEHP [35]. Exposure of equine COCs to 0.12 μM DEHP also impaired nuclear maturation [40]. It should be noted, however, that the phthalate doses used in those studies were mostly associated with acute exposure, i.e., similar to the concentration that we found in the plasma (47.7 ± 8.9 μM) during DEHP exposure. Taken together, it is suggested that MEHP at low or high concentration impairs oocyst nuclear maturation. Given the role of estradiol concentration in oocyte developmental competence [77], an indirect effect of DEHP, through the reduction in estradiol concentration, cannot be ruled out.

In contrast to the clear-cut effect on oocyte meiotic progression, MEHP did not affect CG distribution during maturation. Further analysis revealed differences in CG distribution between mature and immature oocytes regardless of DEHP treatment, suggesting that others cytoplasmic-maturation features, such as cytoskeletal rearrangement, endoplasmic reticulum relocation and mitochondrial distribution, should also be examined; as such effects on oocyte cytoplasmic maturation might further impair early embryonic development. This notion is strongly supported by the findings that although it was the oocyte that was exposed to MEHP during maturation, its deleterious effect carried over to later embryonic developmental stages. In our previous study, exposing bovine COCs to 50 μM MEHP altered the expression levels of *POU5F1* and *ASAH1* in mature oocytes and in the 2-cell-stage embryos developed from the MEHP-treated oocytes [37]. Whether these alterations also occur with the low doses found in the FF in the current study requires further examination.

In summary, we explored the potential risk and carryover effects of acute exposure to DEHP on the ovarian pool of follicles and oocyte developmental competence. Ultrasonography was used to monitor DEHP-induced alterations in growth and development of the preovulatory follicle in association with reduced steroidogenesis and formation of ovarian pathologies. These could be explained by indirect effects via the HPO axis; however, a direct effect on the follicular cell cannot be ruled out. Although the clearance rate of phthalate metabolites from the circulation was relatively high, a residual concentration of MEHP was found in the preovulatory follicle. Based on the ex-vivo model used in the current study (i.e., oocyte maturation in FFs aspirated from control and DEHP-treated cows), it is suggested that a low concentration of MEHP in the intrafollicular microenvironment of the oocyte impairs its ability to undergo maturation, fertilization and further embryonic development.

## Supporting Information

S1 Table Concentration of DEHP metabolites in urine, plasma and milk. (DOCX)
