# Supplementary material for: Carryover Effects of Acute DEHP Exposure on Ovarian Function and Oocyte Developmental Competence in Lactating Cows
Source: PLoS One. 2015 Jul 8;10(7):e0130896. doi: 10.1371/journal.pone.0130896 (PMC4496077; doi:10.1371/journal.pone.0130896)
Supplement: S1 Table — (DOCX) [file pone.0130896.s001.docx]

| MEHP | 2cx-MMHP | 5oxo-MEHP | 5OH-MEHP | 5cx-MEPP | Sample | Day of experiment |
| --- | --- | --- | --- | --- | --- | --- |
| 34.13 | 0 | 2.054 | 8.84 | 32.14 | urineC1 | 0 |
| 52.0 | 0 | 23.58 | 79.33 | 78.35 | urineC2 | 2 |
| 123.23 | 0 | 14.04 | 65.64 | 98.05 | urineC3 | 4 |
| 54.60 | 0 | 6.16 | 37.07 | 60.71 | urineC4 | 11 |
| 14.73 | 0 | 2.73 | 12.92 | 32.14 | urineC5 | 24 |
| 72.57 | 98.70 | 10.95 | 62.92 | 273.05 | urineT1 | 0 |
| 316550 | 15720.8 | 47774 | 154285.7 | 157792.2 | urineT2 | 2 |
| 170079.8 | 8766.2 | 32671.2 | 102244.9 | 90.58 | urineT3 | 4 |
| 1585.5 | 297.40 | 389.38 | 1419 | 1217.9 | urineT4 | 11 |
| 155.56 | 129.22 | 76.36 | 323.8 | 404.22 | urineT5 | 24 |
| 9.34 | 0 | 0.34 | 2.04 | 40.58 | plasmaC1 | 0 |
| 25.50 | 0 | 0.34 | 1.70 | 93.18 | plasmaC2 | 2 |
| 9.70 | 0 | 0.68 | 4.42 | 74.67 | plasmaC3 | 4 |
| 12.21 | 0 | 0.34 | 1.36 | 57.46 | plasmaC4 | 11 |
| 11.49 | 0 | 0.34 | 1.02 | 52.59 | plasmaC5 | 19 |
| 18.68 | 18.18 | 3.42 | 22.10 | 145.77 | plasmaC6 | 24 |
| 12.2 | 0 | 0 | 0 | 0 | plasmaT1 | 0 |
| 38800 | 114.93 | 438.35 | 1073.5 | 733.76 | plasmaT2 | 2 |
| 56580 | 116.88 | 358.90 | 1163.3 | 598.18 | plasmaT3 | 4 |
| 84.42 | 15.90 | 2.05 | 5.78 | 53.24 | plasmaT4 | 11 |
| 30.17 | 11.03 | 0.34 | 1.36 | 32.79 | plasmaT5 | 19 |
| 20.83 | 0 | 0.34 | 2.38 | 43.18 | plasmaT6 | 24 |
| 10.77 | 0 | 0 | 0.68 | 19.15 | milkC1 | 0 |
| 12.21 | 0 | 1.02 | 3.74 | 55.84 | milkC2 | 2 |
| 125.02 | 0 | 1.36 | 6.46 | 77.59 | milkC3 | 4 |
| 4.67 | 0 | 0 | 0.34 | 12.98 | milkC4 | 11 |
| 3.95 | 0 | 0 | 0.64 | 12.33 | milkC5 | 24 |
| 6.10 | 0 | 0 | 0 | 20.12 | milkT1 | 0 |
| 218.43 | 51.94 | 15.41 | 36.39 | 473.05 | milkT2 | 2 |
| 612.56 | 43.83 | 40.75 | 92.85 | 487.01 | milkT3 | 4 |
| 24.78 | 0 | 1.02 | 1.70 | 23.37 | milkT4 | 11 |
| 12.21 | 0 | 0 | 0 | 10.38 | milkT5 | 24 |

**S1 Table 1. DEHP metabolites (nM) concentration found for each experimental day in three collected fraction: urine, plasma and milk in the control (n=5) and DEHP-treated cows (n=4).**
